# “When she goes out, she feels better:” co-designing a Green Activity Program with Hispanic/Latino people living with memory challenges and care partners

**DOI:** 10.3389/fnagi.2024.1401255

**Published:** 2024-06-18

**Authors:** Rebecca K. F. Lassell, Valeria Tamayo, Triana A. Pena, Misa Kishi, Jessica Zwerling, Laura N. Gitlin, Abraham A. Brody

**Affiliations:** ^1^Department of Health and Wellness Design, School of Public Health, Indiana University, Bloomington, IN, United States; ^2^Regenstrief Institute, Indiana University Center for Aging Research, Indianapolis, IN, United States; ^3^Hartford Institute for Geriatric Nursing (HIGN), NYU Rory Meyers College of Nursing, New York, NY, United States; ^4^Department of Art and Art Professions, NYU Steinhardt, New York, NY, United States; ^5^Arthur S. Abramson Department of Rehabilitation Medicine, Albert Einstein College of Medicine, Bronx, NY, United States; ^6^The Saul R. Korey Department of Neurology, Albert Einstein College of Medicine, Bronx, NY, United States; ^7^College of Nursing and Health Professions, Drexel University, Philadelphia, PA, United States; ^8^Division of Geriatric Medicine and Palliative Care, Department of Medicine, NYU Grossman School of Medicine, New York, NY, United States

**Keywords:** Alzheimer's disease, nature-based, community-engaged, Latine, occupational therapy

## Abstract

**Purpose:**

Utilizing a participatory approach, we sought to co-design a 12-week Green Activity Program (GAP) with Hispanic/Latino individuals living with memory challenges and their care partners, local outdoor professionals, and healthcare providers.

**Methods:**

Participants were recruited via convenience and snowball sampling in the Bronx, New York with Hispanic/Latino persons living with memory challenges and care partners, outdoor activity professionals, and interdisciplinary healthcare providers/dementia experts. Co-design occurred iteratively with 5 focus groups and 4 individual interviews lasting 30–90 min and focused on program and research design. Sessions were recorded and transcribed. Utilizing directed content analysis data was coded using a priori codes program design and research design.

**Results:**

21 participants completed co-design activities: (*n* = 8 outdoor activity professionals, *n* = 6 Hispanic/Latino persons living with memory challenges and care partners, and *n* = 7 interdisciplinary healthcare providers/dementia experts). Participant preferences for program design were captured by subcodes *session duration* (30–90 min), *frequency* (4–8 sessions), and *delivery modes* (in-person and phone). Participants' preferred nature activities included group exercise and outdoor crafts [crocheting], outcomes of social participation, connectedness to nature, decreased loneliness, and stewardship were identified. Preferred language for recruiting and describing the program were “memory challenges,” “Hispanic/Latino,” and “wellbeing.” *Referral pathways* were identified including community-based organizations and primary care.

**Conclusion:**

Co-design was a successful form of engagement for people living with memory challenges that enabled participants to help design key elements of the GAP and research design. Our processes, findings, and recommendations for tailoring co-design to engage Hispanic/Latino people living with memory challenges can inform the development of other programs for this population.

## 1 Introduction

The prevalence of mild cognitive impairment (MCI) and Alzheimer's disease (AD) and Alzheimer's disease related dementias (ADRD) is 1.5 times higher for people who identify as Hispanic/Latino than non-Hispanic Whites (Matthews et al., 2019). Hispanic/Latinos have the second highest incidence of AD/ADRD nationally (Chen et al., 2019; Alzheimer's Association, 2023a) and in New York (Noble et al., 2012) and are projected to increase the most among racial and ethnic groups from 430,000 in 2012 to 2,383,000 in 2050 (Alzheimer's Association, 2023a). The Bronx, New York is one of the top three counties in estimated AD prevalence in the United States (Alzheimer's Association, 2023b). The Bronx is 56% Hispanic with 29% of older adults living with low socioeconomic resources (Furman Center New York University, 2019). People who experience multiple risk factors, including minoritized racial and ethnic groups, low socioeconomic resources, social isolation, and depression are among the most vulnerable for developing AD/ADRD (Livingston et al., 2020). These groups are also at heightened risk for poor outcomes including increased neuropsychiatric symptoms (e.g., depression and apathy; Salazar et al., 2017), premature loss of function (Boltz et al., 2021), and care partner stress (Chiriboga et al., 2019). Few interventions and programs exist that have been designed with Hispanic/Latino people living with MCI and AD/ADRD to improve their brain health and stave off or delay cognitive decline. This study sought to co-design a green “nature” activity program with Hispanic/Latino people living with MCI and AD/ADRD and their care partners to promote an active lifestyle and wellbeing utilizing nature activities they enjoy within their homes, neighborhoods, and communities.

There are many terms that may be appropriate to refer to people of Hispanic origin depending on the audience: Hispanic, Latino, Latina, Latine, and Latinx. The terms Hispanic, Latino, and Latina were created by the United States to categorize U.S residents of Spanish and Latin American descent during the census (Miranda et al., 2023). The gender-neutral term “Latinx” emerged in the 2,000's from gender-diverse Latin Americans. Recently, trans Latin activists created “Latine” as an alternative (Noble et al., 2012). Yet, most individuals identify with a national or regional label (e.g., Mexican, Puerto-Rican, and Boricua). When considering older adults' preferences, only 7% of Hispanic/Latinos 65 and older in the United States have heard the term Latinx and prefer Hispanic or Latino (Pew Research Center, 2020). In this study, we asked participants which terms they felt best represented them. Based on participant preference, we use the terms “Hispanic/Latino.” Participants also preferred the term “memory challenges” to describe MCI and early AD/ADRD used herein.

### 1.1 Disparities in dementia care

In the United States, Hispanic/Latino groups experience disparities in dementia care. Hispanic/Latino individuals are also diagnosed with memory challenges later than non-Hispanic whites, and often lack access to dementia care (Aranda et al., 2021; Tsoy et al., 2021). While AD/ADRD is commonly underdiagnosed (Alzheimer's Association, 2023a), Hispanic/Latinos face additional barriers to receiving a diagnosis. Barriers include limited education about AD/ADRD and a higher likelihood of believing that memory challenges are part of normal aging (Arévalo-Flechas et al., 2014). Coupled with the stigma and cultural shame associated with a diagnosis, Hispanic/Latino individuals or their family members often minimize or deny symptoms and keep memory concerns in the family. Hispanic/Latinos provide more care to persons living with memory challenges who have higher neuropsychiatric symptoms (Sink et al., 2004; Salazar et al., 2017) and report higher intensity of caregiving (Friedman et al., 2015; Fabius et al., 2020).

Estimated direct costs for caring for persons living with memory challenges are $169.1 billion with 13% spent on nursing homes. Key predictors of nursing home admission are functional disability and care partner strain (Gaugler et al., 2009; Spillman and Long, 2009), underscoring the need to sustain function for persons with memory challenges so they can live at home for longer. The need for developing rigorous non-pharmacological interventions to address function and engagement is highlighted in the U.S. Health & Human Services' “National Plan to Address Alzheimer's Disease” Goal 1 to prevent and treat AD/ADRD (U.S. Department of Health Human Services, 2020). This need was also recognized at the National Research Summit of Care (Gitlin et al., 2018a) and by the National Academies of Sciences, Engineering and Medicine (National Academies of Sciences Engineering and Medicine, 2021). People living with memory challenges, their care partners, healthcare providers and health systems (Fazio et al., 2018), and researchers (Williams et al., 2010) are searching for sustainable non-pharmacological interventions to support wellbeing and delay functional decline (Williams et al., 2010; Hughes et al., 2013; Regier et al., 2021). Engaging in valued activities can support wellbeing and sustain cognitive and physical function (Hughes et al., 2013). A growing body of research supports the use of activity as a therapeutic with positive quality of life and health benefits (Hughes et al., 2013; Livingston et al., 2020).

### 1.2 Green prescriptions: benefits and challenges

To support wellbeing green prescriptions have been integrated into healthcare systems in New Zealand, Australia, and the United Kingdom (UK) for middle-aged and older adults (Kerse et al., 2005; Hamlin et al., 2016; Elliot and Hamlin, 2018). *Green prescriptions* describe the process of co-designing nature experiences and activities to improve a person's health and wellbeing and emerged in the 1990's (Robinson and Breed, 2019). We refer to *green activities* as any activity that contains the following components identified by Bragg and Leck (2017): (1) meaningful to participants, (2) involving contact with nature, and (3) social engagement. Green activities can include gardening, outdoor exercise, conservation activities (e.g., tree planting and park cleanups), and activities with animals (e.g., dog walking).

Green prescriptions are currently being implemented in 17 countries around the world. They are thought to provide an enjoyable, low cost, low risk, means to sustain activity engagement in daily life and are rapidly growing (Allen and Balfour, 2012; Hamlin et al., 2016; Bragg and Leck, 2017; Polley et al., 2017; Elliot and Hamlin, 2018; Annear et al., 2019; Kruizse et al., 2019). These interventions are particularly beneficial for people who lack access to nature (Allen and Balfour, 2012) or experience lower socioeconomic resources (Rigolon et al., 2021). However, there are many different types of green prescriptions (e.g., exposure to nature, activity-based prescribing: green care, green exercise, and animal-assisted interventions) that are implemented in varied settings and different populations. Benefits include increased physical activity, health, and wellbeing with sustained behavior change for middle aged and older adults (Hamlin et al., 2016; Polley et al., 2017; Elliot and Hamlin, 2018; Robinson and Breed, 2019; Garside et al., 2020; Husk et al., 2020).

Challenges in implementing green prescriptions involve appropriateness of the prescriptions to meet the person's needs, underscoring the need for skillful tailoring (Bergstad et al., 2017). Tailoring is important to address when developing green activity prescriptions for populations living with memory challenges who require additional adaptations and modifications as their function declines. Critiques of current green prescriptions include challenges with referrals in primary care settings, varying terminology, and overlooking minoritized racial and ethnic groups who have been excluded from research. Institutional policies and a lack of available green resources can also contribute to low referral rates and difficulties with participant engagement when implementing green prescriptions as many of these programs grew from a bottom-up approach. There is a need to involve partners from local healthcare organizations and nature organizations when creating green prescriptions to gain institutional and community support and cohesion. Utilizing a collaborative participatory approach to involve all partners (community, outdoor organizations, and healthcare providers) to develop and implement green prescriptions could mitigate challenges with buy-in and cohesion.

### 1.3 Green activities: potential for people living with memory challenges

Green activities can promote an active lifestyle and contain beneficial components of social engagement, nature, and meaningful activities which can support function and stave off decline (Oh et al., 2021; Regier et al., 2021). Engaging in an active lifestyle and reducing inactivity are key modifiable risk factor for cognitive decline and risk of memory challenges later in life (Livingston et al., 2020). Previous research has focused on a single green activity (e.g., gardening, exercise, and animals) for people living with memory challenges rather than a comprehensive green prescription program. Benefits of gardening include improved wellbeing, engagement, social, cognitive function (Mmakp et al., 2020; Oh et al., 2021), and decreased neuropsychiatric symptoms (Noone et al., 2018; Smith-carrier et al., 2019; Zhao et al., 2020; Lassell et al., 2021; Oh et al., 2021). Outdoor exercise has health benefits for people living with memory challenges with improved neuropsychiatric symptoms and function (Lopez-Ortiz et al., 2021). Benefits of animal-assisted interventions for people living with memory challenges are engagement, wellbeing, being in the moment (Wood et al., 2017; Yakimicki et al., 2019; Babka et al., 2021; Lassell et al., 2022), decreased neuropsychiatric symptoms (Nordgren and Engström, 2012, 2014; Yakimicki et al., 2019), use of a person's cognitive capacities, and social interaction (Fields et al., 2019; Wesenberg et al., 2019; Lassell et al., 2021). There is a need to provide a range of green activity options to better meet the diverse preferences, needs, and geographic conditions of people living with memory challenges and their care partners to address some of the barriers to participation.

Despite the benefits of green activities for people living with memory challenges, there are barriers to accessing parks and green spaces. Barriers include the shame and stigma associated with memory challenges (Swaffer, 2015), neighborhood proximity to green space and parks due to redlining (Yang et al., 2023), and park accessibility and walkability (Yu et al., 2023) for older adults who have comorbid conditions or mobility concerns. Hispanic/Latino groups face additional barriers accessing parks in New York City. While Hispanic/Latino and African American and Black groups having higher access to parks and green space in New York City, the parks are disproportionately adjacent to neighborhood disamenities of crime, lack of traffic safety, and noxious land uses (Weiss et al., 2011). Addressing these barriers are crucial when designing the green activities for this population and require creative problem solving from all groups involved (community, outdoor professionals, healthcare providers).

When people living memory challenges are able to safely access parks and green spaces, they may face barriers in participating in the green activity itself. People living with memory challenges present fluctuating levels of function and safety (Matar et al., 2020). This can make it challenging for care partners to support them in engaging in activities they enjoy. For example, a person could remember their walking route one day and have difficulty remembering it the next. There is a need to match green activity demands to a person's context, function, and needs and provide coaching for safe participation in a comprehensive program beyond a written prescription. We use the term “*Green Activity Program* (GAP)” to delineate the program developed in this study which involves the assessment, tailoring, and prescription or “action” plan of green activities for therapeutic purposes, not just a green prescription itself. While there is potential to integrate green prescriptions across disciplines, occupational therapists (OTs) are uniquely positioned to deliver GAP as the skillful tailoring of valued activities are central to their scope of practice (Dorsey, 2021).

For example, an OT could conduct an evaluation to assess a person's medical history, occupational history (meaningful activities, roles, habits, and routines), function, and environment, including safe access to green activities. The OT could co-create a goal with the person and the care partner to participate in a green activity they enjoy: “To promote an active lifestyle and social connection, Mrs. H will increase her endurance to garden from 5 to 30 min over 12 weeks to participate in a local community gardening group.” The OT could tailor the activity to the person's preferences (planting flowers and gardening group) and level of function (starting with 5 min), adapt and scaffold the activity (e.g., increase/decrease the number of steps in directions, verbal and physical cues, and time gardening), and modify the environment [decluttering the space, raised garden bed vs., tabletop gardening, adaptive gardening equipment (e.g., built up garden shovel handle, seed tape for an easier grasp), and level of social support]. The OT could then create a personalized green activity prescription or action plan and coach the person living with memory challenges and their care partner on goal-setting, strategy training for adapting the activities, safety, and fall prevention techniques.

### 1.4 Potential to integrate a Green Activity Program within dementia care

OTs provide one opportunity to integrate the GAP into dementia care as they are embedded within healthcare systems. Medicare and Medicaid cover in-home OT services to support participation in valued activities for people living with memory challenges through skilled home healthcare (Part A) and home-based outpatient visits (Part B; Medicaid.gov, 2024). Research supports the effectiveness of OTs delivering a Tailored Activity Program to people living with memory challenges and their care partners to support their quality of life, function, and reduce neuropsychiatric symptoms (Gitlin et al., 2017, 2018b, 2021). We sought to develop the GAP which draws on the concept of tailored activities and the therapeutic benefits of nature in improving wellbeing and promoting an active lifestyle (Kellert and Wilson, 1993; Kuo, 2015; Shanahan et al., 2019; Nguyen et al., 2023). The GAP builds upon the evidence-based Tailored Activity Program (Gitlin et al., 2017, 2018b, 2021) but differs from it in that activities are focused on utilizing local green resources. Green activities, for the most part, have not been designed with groups facing multiple risk factors for cognitive decline such as Hispanic/Latino groups in low resourced neighborhoods who have the potential to benefit the most. GAP must be designed to be culturally appropriate for Hispanic/Latinos to effectively meet their needs.

### 1.5 What is cultural appropriateness? Why is it important for program design?

Cultural appropriateness involves two dimensions: (1) surface level and (2) deep structure (Resnicow et al., 1999; Huang and Garcia, 2020). Surface level sensitivity involves creating materials and messages to match observable “superficial” characteristics of a specific population. Superficial appropriateness involves language, people (bilingual staff), images, content, music, location, and clothing that are meaningful and culturally congruent. Deep level cultural appropriateness refers to “the values, beliefs, and traditions of a culture within a community” (Resnicow et al., 1999). Hispanic/Latino core values that influence care partners utilization of supportive programming include *familismo* (identity and responsibility to family), *personalismo* (close relationships with others), *respeto* (respect, including older adults), and *dignidad* (dignity and self-respect) (Arévalo-Flechas et al., 2014). Cultural beliefs include being more likely to consider AD/ADRD as part of normal aging and a stigmatizing and shameful diagnosis. This can provide additional barriers for accessing care and may also perpetuate an inactive lifestyle through withdrawal and social isolation. Family is often central in caring for Hispanic/Latino person living with memory challenges. Designing a program with these elements in mind is important for establishing acceptability, adoption, and uptake (Huang and Garcia, 2020). Cultural appropriateness and adaptations are important to identify contextual factors that can support equity in the uptake and reach of evidence-based programs (Parker et al., 2023).

Utilizing a participatory approach, we sought to co-design an occupational therapist-led 12-week GAP program with Hispanic/Latino individuals living with memory challenges and care partners, outdoor professionals, and interdisciplinary healthcare providers. Specifically, we asked:

1. What session duration, frequency, and delivery mode align with the preferences and needs of people living with memory challenges and care partners?
2. How would people living with memory challenges, care partners, and outdoor professionals like to be supported by the OT?
3. What nature activities do Hispanic/Latino people living with memory challenges and their care partners prefer?
4. What outcomes matter to each group?
5. What are preferred recruitment strategies for each group?
6. What are the possible referral pathways and how do healthcare providers see the program fitting into clinical care?

## 2 Materials and methods

This case study utilized participatory co-design and content analysis to design key components of the GAP and research design. The study occurred between March 2023 and August 2023. Findings are reported in accordance with the Standards for Reporting Qualitative Research (O'Brien et al., 2014).

### 2.1 Setting and participants

This study took place in the Bronx, New York. Data analysis occurred in New York City and Bloomington, Indiana. Participants living with memory challenges were recruited using convenience sampling from three local organizations with Spanish and English flyers and in-person outreach events at seven older adult centers in different neighborhoods in the Bronx to sample participants from different Hispanic/Latino groups. A community advisor and research team members who identified as Hispanic/Latino, two of whom were from the Bronx, reviewed recruitment and study materials and tailored the language to appeal to a variety of Hispanic/Latino groups. Outdoor activity professionals were recruited via convenience and snowball sampling through local outdoor and recreation organizations and listservs. Healthcare providers and dementia experts were recruited utilizing snowball sampling through Montefiore Center for Education in Alzheimer's disease and recommendations from the research team.

To be included in the study, persons living with memory challenges were: (1) 60 years or older, (2) identified as Hispanic/Latino, (3) had memory problems or a diagnosis of MCI or AD/ADRD, (4) spoke Spanish or English, (5) Montreal Cognitive Assessment (MoCA) score of 20–25, which characterizes MCI and early AD/ADRD (Pugh et al., 2018), (6) and capacity to provide consent with score of 3–6 using a 3-item standardized approach (Palmer et al., 2005). Participants living with memory challenges underwent initial eligibility screening via phone and a full MoCA screening either in-person or via Zoom in adherence with the MoCA's video conference protocol. Care partners were (1) 18 years or older, (2) spoke Spanish or English, (3) provided mostly unpaid care to the person living with memory challenges. Outdoor activity professionals and interdisciplinary healthcare providers and dementia care experts were: (1) 18 years or older, (2) spoke English or Spanish, (3) member of a local outdoor or healthcare organization, (4) at least 1 year of experience in either green activities or working with older adults with MCI or AD/ADRD. Additional criteria for dementia experts: (5) 4≥ publications related to dementia, (6) leadership position, or 5≥ years working with persons living with memory challenges. Participants were excluded if they did not meet the inclusion criteria. The NYU Langone and Indiana University-Bloomington's Institutional Review Boards approved this study.

### 2.2 Co-design sessions and cultural appropriateness

Surface level cultural appropriateness utilized in this study included language, people (Hispanic/Latino ambassador and team members), images, location (local older adult centers), and preferred method of contact. Specifically, we asked input on terms and language that was inclusive of a broad range of Hispanic/Latino groups and empowering words to describe Alzheimer's disease and memory loss. Additionally, recruitment strategies accounting for location and preferred method of outreach such as in-person events or emails were also included. Deep structure appropriateness during this study included offering options to deliver activities that related to *personalismo* (e.g., community gardening, walking group) and *familismo*, asking to include family in the delivery of the program. To design GAP to be culturally appropriate, we applied co-design.

Co-design is defined as “the meaningful end-user engagement” in all stages of the research process with a range of intensities from passive to highly involved (Slattery et al., 2020). While co-design methods range from individual interviews to town halls, participatory co-design as applied in this study emphasizes rapid-cycle design iterations with a smaller group (e.g., ~5 people; Slattery et al., 2020; Werner et al., 2022). There is limited understanding of how to tailor co-design for older adults and people with disabilities (Slattery et al., 2020), including people living with memory challenges. Co-design can be useful for asking more relevant research questions for community and organizational partners, more effective knowledge translation, and quicker uptake of evidence into practice (Nilsen, 2013). This study provides an example of how to design a program with input from multiple partners including the end-user Hispanic/Latino people living with memory challenges who are disproportionately impacted by AD/ADRD and are often overlooked in research. Our findings and co-design process can be useful to other researchers designing interventions and programs for this population.

Research team members received training in dementia friendly approaches and moderating focus groups. Trained research members (VT, TP) identified as Hispanic/Latino and spoke Spanish and English delivered co-design sessions. Group co-design sessions were delivered with a trained moderator and a note-taker with focus group methodology. An effort was made to conduct co-design sessions with 4–8 participants based on best practices for focus groups (Krueger and Casey, 2014). For Hispanic/Latino people living with memory challenges, we sought to have smaller groups to be less overwhelming and avoid distractions (Hall, 2020). An ambassador who identified as Hispanic/Latino, spoke Spanish, and had parents living with memory challenges greeted participants to help them feel comfortable. Individual co-design interviews were offered for participants who were unable to attend group co-design group sessions based on recommendations for flexible engagement options to support the inclusion of people living with memory challenges in research (Griffith et al., 2023).

**Figure 1:**
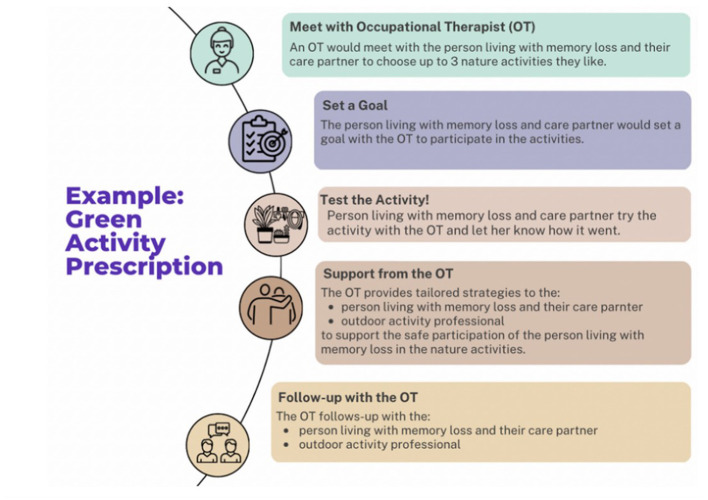
Initial prototype of the Green Activity Program.

Co-design sessions were scheduled based on participant's availability and were delivered in-person for Hispanic/Latino persons living with memory challenges at their local older adult centers and virtually via Zoom for outdoor professionals and healthcare providers/dementia experts based on their preferences. Sessions occurred in the language of participants' choosing and ranged from Spanish, Spanglish, and English. Co-design occurred using a semi-structured topic guide consisting of topics: preferred nature activities, session frequency, duration, delivery mode, outcomes, recruitment strategies, and referral pathway(s). Co-design sessions began with open ended questions, “What nature activities do you enjoy?” followed by more focused questions “How often should sessions occur: weekly, every other week, a range of 4–8 sessions?” using an initial prototype of the program (Figure 1). The prototype was based on the feasible and effective Tailored Activity Program Home version for people living with dementia (Piercy et al., 2009; Gitlin et al., 2021) and two successful green prescribing models namely, New Zealand's Green Rx (Elliot and Hamlin, 2018) and the United Kingdom's social green prescribing (Bragg and Leck, 2017). The prototype was iteratively refined after each co-design session based on participants' feedback.

### 2.3 Data collection

Descriptive and qualitative data were collected during the study. Descriptive data included the MoCA and demographic data. Trained team members (RL and VT) administered the MoCA either via Zoom or in-person based on participant's preferences. Study data was collected, de-identified, and managed using REDCap electronic data capture tools hosted on an NYU Langone secure server. Qualitative data consisting of audio recordings and field notes were collected. Field notes were captured during co-design sessions to document the conversation and co-design processes (Glesne, 2016). Interviews were completed in a private space, audio recorded, transcribed and translated by a professional transcription service and reviewed for quality by VT and RL. VT verified and updated any inconsistencies or errors in translation from Spanish to English. Participants were identified by their participant category and a number to provide confidentiality (e.g., care partner 1, person living with memory challenges 2, etc.). The transcripts and field notes were reviewed for quality (VT and RL) and uploaded into NVivo, a qualitative data analysis software (www.qsrinternational.com/nvivo/home).

### 2.4 Data analysis

Two trained team members (RL and VT) analyzed the transcripts and field notes using directed content analysis. Directed content analysis is a type of content analysis focused on the systematic categorization of content to identify trends and patterns of words with low interpretation (Hsieh and Shannon, 2005). Content analysis is applied “to explore who says what, to whom, and to what effect” (Vaismoradi et al., 2013). Directed content analysis in this study was rooted in Communication Theory and a factist perspective with the assumption that data can be an indicator of a person's reality (Sandelowski, 2010) and can be used to understand their attitude, behavior, and motives. Directed content analysis is a deductive approach that begins with a priori codes derived from key concepts or research but allows for new codes to emerge (Hsieh and Shannon, 2005).

We applied directed content analysis to capture content relevant to the concepts of program and research design to create the GAP and design a future feasibility study. Start codes were *Program Design:* preferred nature activities, session frequency, duration, delivery mode; and *Research Design*: preferred outcomes, recruitment strategies, and a referral pathway. Utilizing Hsieh and Shannon's iterative process (Hsieh and Shannon, 2005), VT and RL (1) independently read through each transcript and coded based on pre-established codes, (2) compared codes, and (3) created new codes until saturation was met. When comparing codes, VT and RL discussed coding disagreements by providing a rationale based on the definition of the code, examining the context of the quote, and discussing until consensus was reached. A third team member was available as a tie-breaker if consensus could not be reached. New sub codes that emerged were *initial thoughts and potential barriers and solutions*, and a new code *Main-takeaways*.

#### 2.4.1 Quality, trustworthiness, and reflexivity

Four methods were utilized to support quality and trustworthiness: an audit trail, member-checking, triangulation, and negative case analysis (Savin-Baden and Major, 2013). An audit trail was used to document key decisions that were made in the coding process in Nvivo. Member-checking was conducted at the end of each co-design session by TP or RL to summarize participants' perspectives and verify accurate representation. Triangulation involved checking codes across data points (transcripts and field notes) to support consistency. Negative case analysis was used to identify conflicting perspectives.

RL identified as a female, cisgender, non-Hispanic, and white person. RL reflected on how her identities influenced the research process and discussed often with coauthors and team members throughout the study. RL created a research team (VT, TP, and JY), an ambassador (CT) and a community advisor (AT) who identified as being from Hispanic/Latino groups with three members who were from the Bronx to refine recruitment materials and co-design sessions to be culturally sensitive. For example, team members replaced a focus group moderator technique of looking away as a non-verbal cue to stop speaking and replaced it with a group signal (e.g., raising hand) to align with the Hispanic/Latino value of *respeto*, particularly for older adults. RL was intentionally absent during Hispanic/Latino co-design sessions which were led by team members VT and TP with ambassador CT to increase participants' comfort in sharing their perspectives. RL completed data analysis with team member VT to support accurate representation of the results.

## 3 Results

Twenty-two eligible participants completed consent with *n* = 21 completing co-design with *n* = 4 people living with memory challenges and two care partners who identified as Hispanic/Latino (*n* = 6 total), *n* = 8 outdoor professionals, and *n* = 7 interdisciplinary healthcare providers. Due to small sample sizes in each group, we present demographic findings generally to protect participants' anonymity.

Thirty-four people living with memory challenges and care partners indicated interest with *n* = 11 meeting initial eligibility (*n* = 7 people living with memory challenges, *n* = 5 care partners). One person living with memory challenges and one care partner did not respond to follow-up, and one care partner declined participating, due to an unexpected life event. Six people living with memory challenges were screened using the MoCA and four met eligibility. All four were assessed for capacity to provide consent. All four were eligible, enrolled in the study, and completed co-design. Three care partners enrolled in the study, one did not respond to scheduling attempts, and two completed co-design. People living with memory challenges were mostly female (75%), mean (M) age: 67, spoke Spanish (50%) and Spanish and English (50%), most had college or some college education (75%), and lived alone (75%). People living with memory loss and care partners were from Ecuadorian, Mexican, and Puerto Rican origins. Care partners were female and provided care to a parent living with memory challenges. MoCA scores for people living with memory challenges ranged from 22 to 25 (M:23). Quotes are presented as spoken (e.g., Spanish, English, or Spanglish) with verified translation afterwards.

Nine outdoor activity professionals indicated interest, all met eligibility. One did not respond to follow-up. Eight outdoor activity professionals enrolled in the study and completed co-design. Outdoor activity professionals worked mostly for non-profit organizations (62%) with professional backgrounds in nature-based art making, eco-gerontology, physical activity, volunteer landscaping, horticulture, and greenway stewardship. Most were female and non-Hispanic (75%). Outdoor professionals identified as White (50%), African American or Black (12%), and American Indiana or Native Alaskan (12%). All spoke English and half worked with people living with memory challenges. Experience providing outdoor activities ranged from 2 to 25 years (M:10 years).

Seven healthcare providers and dementia care experts showed interest, were eligible, and enrolled in the study. Healthcare providers and dementia experts represented diverse disciplinary perspectives of a geriatrician, neuropsychologist, two occupational therapists, a psychiatrist, and two social workers. Nearly half were clinician-scientists (43%) in a local academic health system. The group was female and identified as non-Hispanic and spoke English (85%) and Hispanic/Latina and spoke Spanish and English (15%). Providers and dementia experts identified as White (57%) and African American or Black (29%). All worked directly with people living with memory challenges, mostly with racially ethnically and socioeconomically diverse patients in the Bronx, NY (85%). Experience ranged from 4 to 16 years (M:10 years).

**Figure 2:**
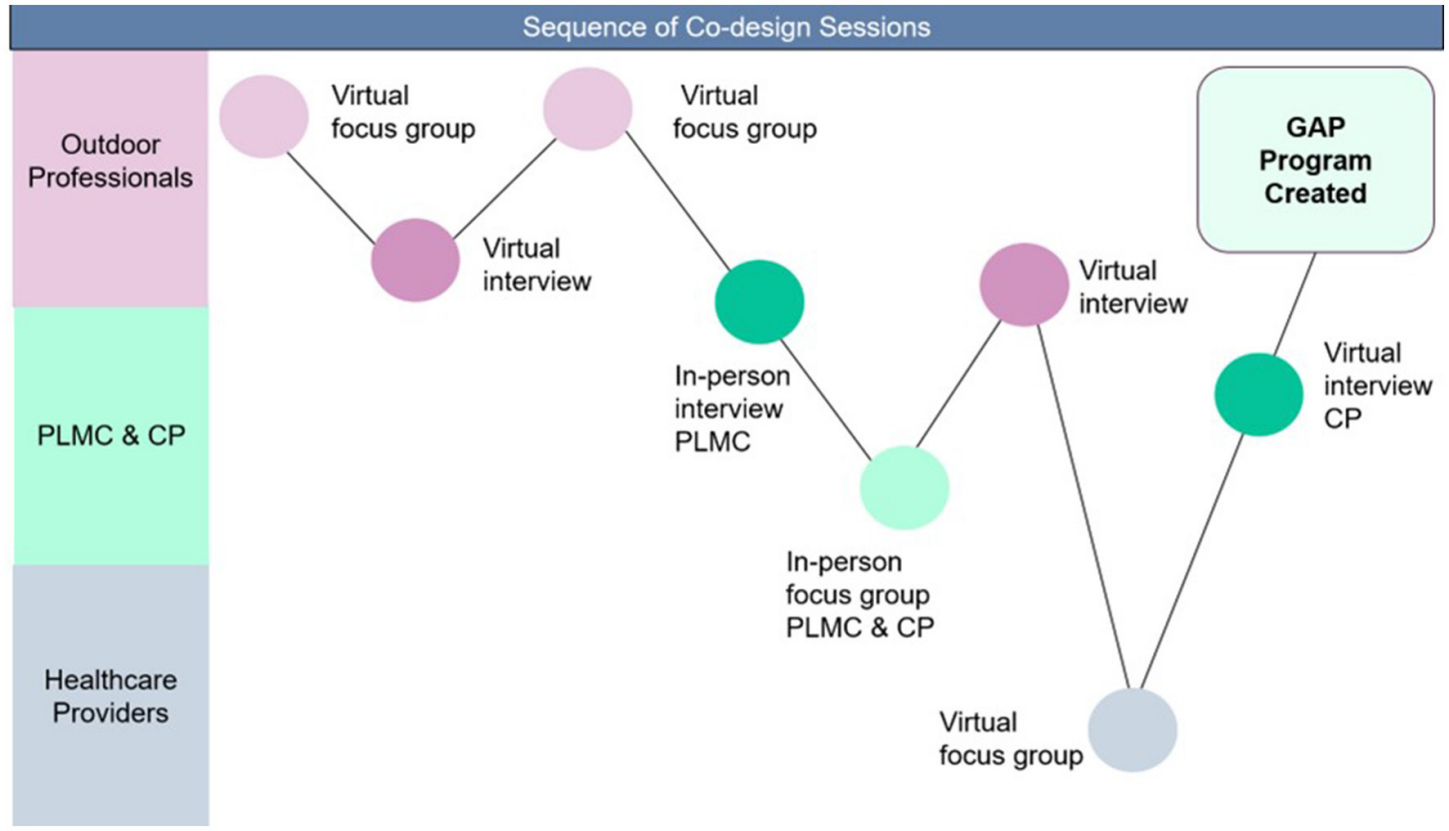
Sequence and type of co-design session. CP, care partner; GAP, Green Activity Program for Hispanic/Latinos; PLMC, people living with memory challenges.

Co-design occurred in the following sequence with a mixture of group discussions and individual interviews to be inclusive of participants' needs and situations (Figure 2).

Two main codes *Program Design* and *Research Design* with new subcodes *Initial Thoughts, Potential Barriers and Solutions*, and code *Main Takeaways* that emerged. Results for Hispanic/Latino people living with memory challenges are presented as they were spoken in Spanish, English, or Spanglish.

### 3.1 Program design to guide initial development

*Program design* described content related to developing the program and included subcodes*: initial thoughts, session duration, frequency, delivery modes, support from the OT, and preferred green activities*. Participants providing input on the design of the program included (1) Hispanic/Latino people living with memory challenges and care partners and (2) outdoor activity professionals.

#### 3.1.1 Initial thoughts

*Initial thoughts* described initial reactions to the GAP. All people living with memory challenges and care partners reacted positively to the idea of the program. A person living with memory challenges said, “A mi me gustó la idea. La siento bien positiva, bien el contacto con la naturaleza.” [*I liked the idea. I feel very positive, [about] good contact with nature.]* When asked about the name “Green activity Prescriptions” there was initial disagreement. One care partner said “It is very professional. It sounds really nice and not intimidating.” The care partner clarified, “I think it's a good idea depending on if you're comfortable with prescriptions.” Participants discussed the possibility of doing nature activities indoors and reached consensus on keeping the name but suggested providing an explanation as provided in the focus group.

**Figure 3:**
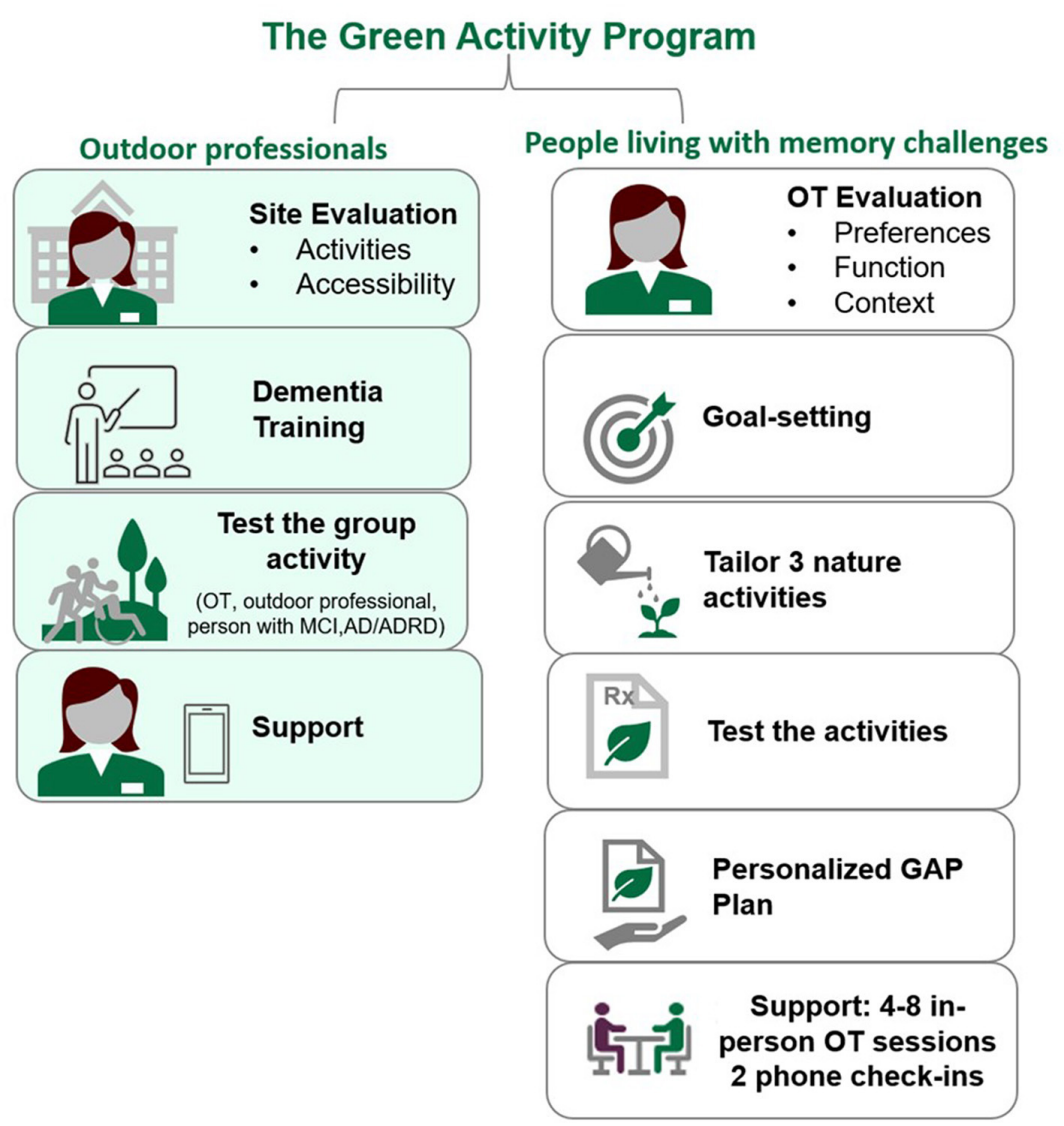
Key components of the Green Activity Program for Hispanic/Latino people living with memory challenges.

Most outdoor activity professionals liked the idea initially and felt the program was very “person-centered,” while one professional did not provide an opinion. One outdoor activity professional suggested adding a training component for outdoor activity professionals. “What you'd want to maybe have the OT do [for outdoor professionals] some education on, you know, working with people living with dementia…so that those that are running the outdoor education, or the restoration projects have…dementia friendly awareness.” This idea was confirmed by all outdoor professionals. “I just don't have enough knowledge about what the best practices for preparing folks to work with folks with dementia…” another outdoor professional said. In response, we added a separate arm of the program and included dementia training and strategies to safely modify activities (Figure 3).

#### 3.1.2 Session frequency, duration, and delivery mode

This subcode described how often (*frequency*) people living with memory challenges, care partners, and outdoor activity professionals would like to receive program sessions, for how long (*duration*), and how they would like to receive sessions (*delivery mode*).

All people living with memory challenges agreed upon a range of 4–8 in-person sessions lasting 30–90 min with the OT, and 20–30 min phone-check-ins. The frequency and duration was confirmed by all care partners. People living with memory challenges and care partners emphasized the importance of flexibility and tailoring when considering *session duration* and *frequency* based on the person's situation and how they may be feeling. A person living with memory challenges said, “Que fuera flexible.” [“*Let it be flexible.”]* A care partner shared the need for flexibility based on a person's situation.

It will be no issue meeting you every week. But for some other people, they might say, oh, I couldn't get [to] the activity today. I couldn't do this. So I think maybe like 2 weeks and then once on the phone for a few minutes, I guess to discuss whatever it would be, it would be good, I think.”-Care partner

Another care partner relayed the need for flexibility based on the complexity of coordinating work and time spent caring for their family member living with memory challenges.

Entonces ahí yo tendrían más disponibilidad de tiempo y diría así la quiero hacer una vez a la semana o cada quince días o o sabe que sólo los sábados depende de cuántas horas me den, porque yo sólo tengo tres horas con el. *[Once a week or every 15 days, you know that only on Saturdays it depends on how many hours they [work] give me, because I only have 3 h with him.]*-Care partner

Both perspectives highlight the need to tailor the GAP to each person's needs with flexibility around session frequency and duration.

People living with memory challenges and care partners agreed upon offering a range of program *delivery modes* including in-person sessions, virtual, and phone consultations. Yet, all preferred in-person and phone check-ins. There was differing levels of comfort with technology and virtual sessions. Half of the people living with memory challenges did not want virtual options, highlighting the need for personalized choice. One care partner suggested hybrid group sessions where “you could have a couple of people on Zoom and the other ones are not into Zoom.” However, one indicated they were willing to try virtual sessions: “I'm not very good with technology and the tablet, but, I can learn how to use it.”

Outdoor activity professionals agreed on a *session frequency* of 2–4 visits over the 12-week program. Professionals requested an in-person onsite evaluation and training first to support people living with memory challenges in their programming, one in-person visit with the OT and the person living with memory loss to practice the strategies and offering check-ins for a *duration* of 30–60 min that could be personalized based on need.

I think the consultation in-person would be especially important at the beginning both to go to the specific project sites, but also to potentially meet with other staff members that would be working with the participants and some of the kind of prep for working with the participants… by a professional. *-*Outdoor activity professional.

#### 3.1.3 Support from the OT

*Support from the OT* described how people living with memory challenges, care partners, and outdoor activity professionals would like to be supported by the OT outside of program sessions. People living with memory challenges varied in how they wanted to receive support from the OT ranging from paper handouts only with most requesting multiple modes of support: text messages, demonstration videos, and paper handouts. All agreed that it was important to be able to contact the OT directly with questions. Care partners also differed in how they wanted to receive support from the OT from text messages to having a range of options (e.g., text messages, demonstration videos, and paper handouts) underscoring the importance of honoring personal choice.

All outdoor activity professionals also agreed on the importance of having a range of options for support from the OT throughout the program. There were also differences in preferences for receiving support ranging from in-person support only Zoom or phone communication with written strategies, no response, with over half requesting a range of options including video demonstrations, text messages, and paper handouts. All wanted to contact the OT via phone if an issue arose.

#### 3.1.4 Preferred green activities

*Preferred green activities* described nature activities that participants recommended or enjoyed. A list of local nature activities was first refined by outdoor activity professionals and then reviewed by people living with memory challenges and their care partners. Outdoor professionals recommended structuring activities “by organization and site,” for organizations who were interested and gave permission to participate. Outdoor professionals shared several organizations and listed specific activities of outdoor yoga, accessing local green spaces, sailing, and a rainbow garden. They also brainstormed nature activities and programming that could be done virtually or indoors in inclement weather. Outdoor professionals indicated outdoor crafts like “birdhouse building” and make “a seed ball” with native plant seeds could be done indoors during inclement weather. Another outdoor professional conducted virtual programming focused on care partners living at home:

I do a lot of educational programming online, training for care partners in green, in nature, awareness activities and things so that can be done year round at home… And my work does involve educating care partners on how to grow and nurture nature connectedness daily in their day to day practice at home. -Outdoor activity professional

People living with memory challenges who identified as Hispanic/Latino first shared outdoor activities they enjoyed, which included walking, bird watching, feeding the birds and squirrels, gardening; outdoor exercise; outdoor music, arts and crafts (crocheting, painting); and cultural activities (cooking cultural recipes, festivals).

... a mi me me encanta el gardening y bueno, ahora mismo bailar afuera, en la playa Yen parques. *[…I love gardening and well, right now dancing outside on the beach and in parks… It's two activities that I love to do.]*-Person living with memory challenges

Most new activity suggestions had social undertones acknowledging the value of *personalismo*.

My mother is more sedentary because she's 85. So an outdoor activity where you have a different… park outdoor area where maybe some of them might engage into, I don't know, uh throwing something or painting or watching someone else doing something that they could intervene and, and do things… like for her because she's from Mexico, maybe an activity that they have to do with recipes and things, like share your idea and your family and culture or, you know, people who bring their own culture, ideology into place um doing activities that [they] model with their hands. *-*Care Partner

Another person living with memory challenges expressed a desire to participate in the green activities with another person.

Lo mas que me gustaría es caminar con alguien y conversar y la otra es de sembrar arboles y limpiar. *[The most I would like is to walk with someone and talk and the other is to plant trees and clean.]*-Person living with memory challenges

This idea was confirmed by a care partner who shared the importance of group activities for her mother, but also for herself: “I'm gonna push her to the things, but it's good that they are with other people. And I realize I'm not alone.” However, when asked directly if participants wanted to do the activities with family most people living with memory challenges said “no,” yet half would like to do green activities with friends, highlighting the need for tailoring.

After participants shared their favorite green activities, the facilitator read the list of local green activities that were co-designed with the outdoor activity professionals. Participants were asked to add to the list and identify non-preferred activities. Participants with memory challenges added sightseeing and a care partner added outdoor meditation. There were differing preferences for fishing (half disliked another liked), it was unanimous that virtual programming was non-preferred, with one voicing dislike for composting,

### 3.2 Research design to inform a future feasibility study

*Research Design* codes described content related to the design of a future feasibility study and included subcodes*: outcomes, recruitment, referral pathways*, and *potential barriers and solutions*. All participants provided input on all codes except *referral pathways* which was designed by healthcare providers.

#### 3.2.1 Outcomes

*Outcomes* described what participants wished to gain from the GAP. Outdoor activity professionals were shown a list of potential outcomes and added stewardship (number of seeds planted, trees stewarded), program attendance, the person living with memory challenges' experience, and changes in behavior. They also expressed the importance of collecting outcomes utilizing mixed methods, particularly interviews, and tracking outcomes related to place and intergenerational connections. “We primarily work with college and high school age students. So being able to, you know, incorporate those folks [youth] into the outcome as well. Making those kind of intergenerational connections might be something,” said an outdoor activity professional who noted older adults tended to be the “historians” of the neighborhood.

All people living with memory challenges agreed on the importance of learning and sharing with others and feeling connected with nature. A person living with memory challenges said, “Conectarme má s con la naturaleza y también conocer a nuevas personas, muy agradable.” *[“Connecting more with nature and also meeting new people [would be] very nice.”]* Both care partners agreed.

la conocer de otras personas engobla todo…Y al mismo tiempo salir de ese espacio ósea de salir de desconectarse, de, de de estas preocupaciones, de de eso que uno tiene. –*[knowing other people encompasses everything… And at the same time, get out of that space, get out of disconnecting, from, from these worries, from what one has.]*-Care partner

This perspective highlights the need for social connection, reducing social isolation, and a change of scenery.

Interdisciplinary healthcare providers and dementia care experts provided input on screening tools, demographic data, the occupational therapy evaluation, and pre/post outcome measures. Providers emphasized the importance of adding demographic measures to describe the population with a focus on social determinants of health. One voiced against providing a functional cut-off measure for study inclusion criteria:

…part of the wonderful thing about this [GAP] is that, regardless of where you are on your functional status, you should be able to engage in some sort of outdoor activity, and that's going to be part of the burden of the OT is to figure out what's like feasible and what's realistic, given how they are functioning… but I don't know that the functional measure needs to be something that tells you, “Oh, either you're able to participate, or you're not.” It maybe gives a better picture of what are realistic goals to be set and options for this participant. -Healthcare provider

Healthcare providers also suggested measures of loneliness and sleep and suggested assessing mood, anxiety, and depression using the Geriatric Depression Scale instead of the Patient Health Questionnaire-9 (PHQ-9). They also recommended using the telephone Montreal Cognitive Assessment (T-MoCA) to screen for cognition with low participant burden. In response, we added demographic measures and included the CMS' Health Related Social Needs tool to screen for social determinants of health.

#### 3.2.2 Recruitment

*Recruitment* described preferred recruitment strategies, contact methods, and empowering language for recruitment materials. People living with memory challenges and care partners provided guidance on *recruitment strategies*. Specifically, strategies to build trust were in-person outreach events at the local older adult centers with flyers and follow up via phone calls with options for text messages. People living with memory challenges and care partners identified *representative terms* for recruitment materials. They agreed that “Hispanic/Latino” best represented them among groups instead of Latina, Latinx, or Latine. They also recommended *empowering language* by using the term “People living with memory challenges” instead of “people living with dementia,” “Alzheimer's disease and related dementias,” “memory loss” or other terms in the community. We updated language from “memory loss” to “memory challenges” on the prototype of the program as a result. However, they preferred having their healthcare providers use exact terminology in the clinic (e.g., Alzheimer's disease and vascular dementia). There was unanimous agreement among people living with memory challenges and care partners in using the term “wellbeing” to describe the main outcome of the program on recruitment materials. Outdoor activity professionals and health care providers preferred recruitment methods were email.

#### 3.2.3 Referral pathways

**Figure 4:**
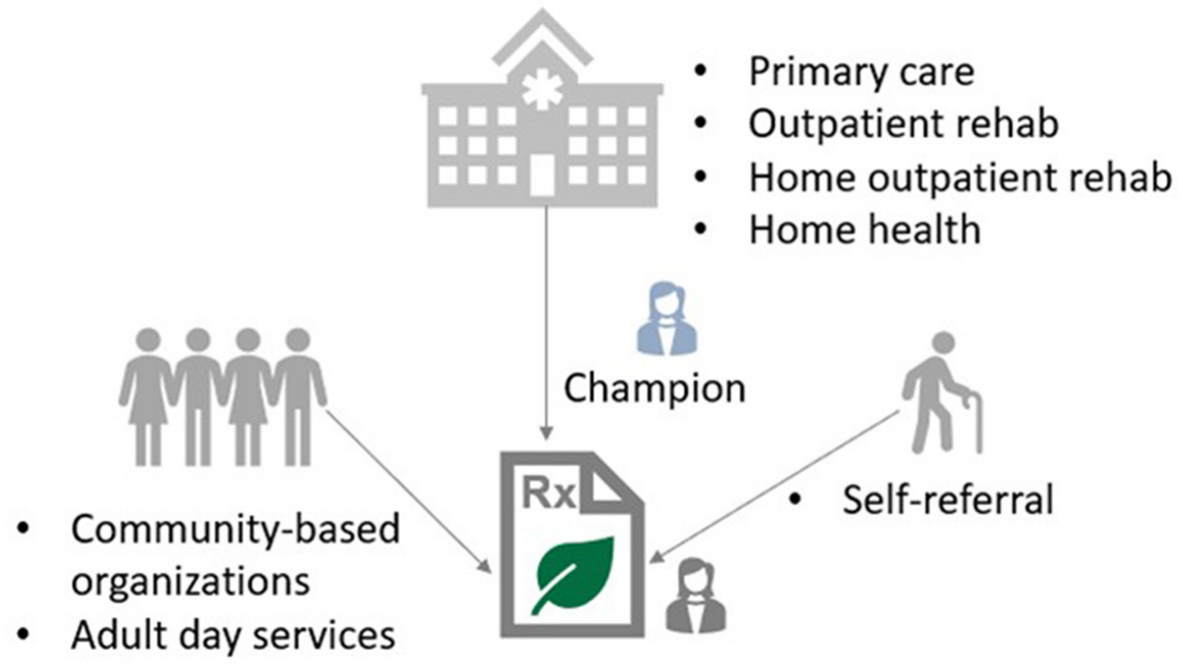
Referral pathways to the Green Activity Program.

*Referral pathways* described settings and processes where people living with memory challenges and their care partners could connect to the program and how it may fit within clinical care (Figure 4).

Interdisciplinary healthcare providers recommended several settings where referrals to the GAP could fit into clinical care. One provider recommended “outpatient in the home,” in addition to home healthcare and traditional outpatient services. Others recommended community-based organizations and adult day services. Another recommended primary care but highlighted some of the benefits and challenges:

…primary care doctors are stretched extremely thin and patients who have cognitive impairment, the cognitive impairment is not being recognized by at large…which is unfortunate, and I think primary care doctors again sometimes feel some nihilism that there's nothing to do. So, you know, giving more tools in the toolbox would be really helpful. But we have such a gap in terms of even recognizing cognitive impairment in the first place. So although I'm sad to see this, maybe relegated to once someone already gets diagnosed. I think that's where it's more likely to be more actively used at this stage of the game… -Healthcare provider

Referral processes discussed included a referral champion in the clinic like a “nurse” or “social worker,” interdisciplinary healthcare providers who were medical doctors liked the idea of having a paper prescription pad as a cue for referrals. Another suggestion was adding a social worker as a team member if budget allowed to address health related social needs.

You might really need to have like a social worker on your team, because…if the person maybe does [have] the insurance, they might have access to more support than they actually realize. Maybe they have a long-term care program that might be able to give them some assistance with getting around. So there may be more options than they know, but you would need a kind of an advanced social worker to be able to look into that. -Healthcare provider.

There was consensus as a group that adding a social worker would be important if possible. A social worker or psychologist on the team was also requested by one care partner to address navigating relationships and behaviors. Another healthcare provider highlighted the importance of the OT in breaking down barriers to participation related to social determinants of health with the suggestion of creating goals to both access and participate in the green activity.

I would be interested to see what people's goals are, and if OT feels a need more to work on ways to accommodate or adapt the way that they're getting to the activity vs. actually participating in the activity, because I know the goal is to be in the community, to be outside. But if your goal is to…look at the flowers in the Botanical Garden, but you have to take a bus to get there. Then me as an OT, I'm thinking…How do we work to manage that part of the activity vs. engagement in the activity itself, because it seems like a prerequisite to get there…. -Healthcare provider

#### 3.2.4 Potential barriers and solutions

*Potential barriers and solutions* described possible challenges and problem-solving solutions that emerged from co-design across all three groups of participants. Potential barriers to participating in the green activities identified across groups included transportation, food insecurity, and safety. Potential solutions were utilizing community resources for discounted or free transportation for older adults (e.g., Access-a-ride, reduced public transportation fare), including social work, and utilizing OT goal setting to access the activity safely and wearing a bracelet with emergency contact information.

I also think the OT on the team might play a role in helping to break down some of the barriers depending on what those barriers are. [The OT] may be able to help support their engagement in [the] activity, or it might be like a reframe… It might be that some individuals we talk about one activity, but if there's X, Y, and Z barrier we talk about, okay, can we bring that activity to you? -Healthcare provider

Other challenges identified were cultural differences, language barriers, and seasonal activities. One solution identified for cultural differences was asking people's preferences for communication, how they would like to be addressed and greeted. Participants suggested having an interpreter or community health worker translate during sessions for language barriers during group activities. Outdoor activity professionals also generated a list of indoor/year-round nature activities offered in the community with virtual options that could be accessed from home.

### 3.3 Main take-aways

*Main Take-aways* were closing thoughts or comments. For people living with memory challenges and their care partners, main takeaways included learning from others and about green activities.

…what they are trying to implement, believe it or not, is an immense help for families. Because they are helping them remember… Today he didn't recognize the apartment. But these activities help. I see that when she goes out, she feels better, she shares, and she talks more. Because at home he looks at the floor all day and wants to go out. -Care partner

A person living with memory challenges shared: “For me is more information and sharing with people that I feel comfortable.” One person also expressed the need for competent supervision.

Pues lo mas importante como te digo seria el que pue que uno tiene con quien caminar en el parque, o pasear en una vereda bonita, observar. Porque hablaba que yo voy a necesitar el programa ya va a ser en una edad que no voy a poderlo hacer sola. *[Well, the most important thing…would be that you have someone to walk with in the park, or walk on a beautiful path, to observe. Because I was saying that I'm going to need the program at an age where I won't be able to do it alone.]*-Person living with memory challenges

He also shared that family typically assist and highlighted the shortcomings of paid care, stating that “... usualmente que nos envían, que cuidan los ancianos son un desastre” *[“…usually those who [they] send us, who take care of the elderly are a disaster.”]* This quote highlights a general need for improved quality of care and problem solving with families and people living with memory challenges to provide competent supervision during the program.

Outdoor activity professionals expressed a range of main take-aways from being “impressed with the depth of the study” and appreciation for “seeing them [participants] as individual” and “giving them the power.” Two outdoor activity professionals expressed the need to achieve buy-in and align outcomes with organizational goals.

I think there are lots of people who will be really excited to be involved in this process but may jump out after that initial consultation with the OT, [but] if it doesn't quite align with particular outcomes [of the organization] it could be a challenge. -Outdoor professional

The outdoor professional also highlighted the importance of being “as flexible as possible with what those outdoor activities look like” as lots of organizations providing nature programming are focused on youth. This led to more discussion about interest in exploring “intergenerational opportunities” for nature programming from three outdoor professionals who highlighted both older adults and youth are experiencing deprivation from nature.

*Main Take-aways* for healthcare providers were positive. A healthcare provider said, “I think you really have the potential to improve quality of life.” One suggested distilling the population of focus with another suggesting focusing on people who are inactive with a mixture of physical and mental health challenges. She noted that it could be “incredibly powerful,” stating “I'm wondering if there's a way to target those individuals who really need that additional connection.” In response, we added measures of sedentary behavior in addition to mental health measures in the feasibility study and are focusing recruitment through community-based organizations to have a broader reach. Another healthcare provider shared, “It's innovative. I think that this actually has a lot of potential” and expressed a desire to be involved after the feasibility study.

After the healthcare provider design focus group, we finished with an individual interview with a care partner to review final changes to the program and research design.

## 4 Discussion

We co-designed the GAP with Hispanic/Latino people living with memory challenges and care partners, outdoor activity professionals, and interdisciplinary health care providers. This study is innovative in that it provides an example of community engaged research utilizing co-design with more than one group of partners and is one of a few that includes Hispanic/Latino people living with memory challenges in the design process. Findings underscore the need for flexibility and tailoring when working with people living with memory challenges and also the need to provide capacity building for local nature organizations who are interested in including them in their programming. The process and findings from this study provide an example that can guide other community-engaged researchers seeking to collaborate with multiple types of partners, including people living with memory challenges to design programs to meet their preferences and needs.

Our finding that co-design was a successful form of engagement for people living with memory challenges at the early stages builds on previous work calling for their inclusion in research (Tanner, 2012; Littlechild et al., 2014; Griffith et al., 2023). Our findings highlight the importance of flexibility in including people living with memory challenges in research and aligns with Griffith et al. (2023). Specifically, we utilized flexible methods for participation with options for individual interviews. While it is ideal to keep uniformity of interview methods, strict methodological rigor can result in the exclusion of people living with memory challenges. Griffith et al. (2023) states, “Flexible attendance/participation also allows individuals with more advanced symptoms to continue participating and contribute insight from a rarely included perspective” (p. 4). Participants also requested flexible participation in the GAP which was built into the final design of the program with options for frequency and duration based on need. As the field advances in including people living with memory challenges in research, flexibility will be crucial in our ability to include and collaborate with them to meet their needs.

Co-design enabled us to design the GAP to align with cultural preferences and values of Hispanic/Latino people living with memory challenges and their care partners. Deep structure cultural sensitivity of the program occurred with alignment in cultural values with the range of nature activities that were selected. Core cultural values of *personalismo*, and *dignidad* (Arévalo-Flechas et al., 2014), particularly pride and self-respect for culture were apparent in preferred activities of walking with a friend, enjoying traditional food and celebrating together (e.g., dancing on the beach, cultural music, food, and events). Interestingly, most participants with memory challenges did not want to do green activities with family members, which relate to the values of *familismo* and *dignidad*. Perhaps, participants with memory challenges were concerned about being a burden to their family members and prioritizing their needs over their own as many lived alone, or maybe they desired dignity in maintaining independence in green activities. Participants were open to engaging in green activities with others, which could address social isolation, a modifiable risk factor for cognitive decline and AD/ADRD (Livingston et al., 2020). Participants also provided input on terms they felt best represented their communities (Hispanic/Latino), which aligned with the Pew Research Center's (2020) report. It is important to acknowledge that culture, race, and ethnicity are one of the many identities a person holds. Caution must be taken not to generalize these findings to all people who identify as Hispanic/Latino as a person's values and preferences may vary based on any number of identities (gender, social roles, age, geographic location, religion, etc.). Co-design findings provide a general starting point for culturally appropriate design by identifying a range of preferences that can be personalized based on each family and person's situation.

Hispanic/Latino participants also provided guidance on surface level cultural sensitivity and agreed upon the term “memory challenges” as their preferred term for Alzheimer's disease and related dementias in the community with specific terminology for the clinic. This aligns with cultural values of *respeto* and *dignidad*, particularly around preserving self-respect and avoiding shame and stigma (Arévalo-Flechas et al., 2014). “Memory challenges” was perceived as a neutral term that supported dignity and respect. Notably, nomenclature around AD/ADRD is challenging across cultures. There is disagreement and controversy in the field about which terminology to use in different situations. People living with early onset Alzheimer's disease have been described using seven different terms over the past two decades with “young onset dementia” being the most common (Van De Veen et al., 2021). Healthcare service professionals often omit terminology related to AD/ADRD or utilizes euphemisms to protect the person/family, avoid conflict, and stigma associated with the diagnosis (Hansen et al., 2016). Moreover, family care partners of people living with memory challenges often use terms to describe neuropsychiatric symptoms that are differ substantially from clinical terms (Gilmore-Bykovskyi et al., 2020). This underscores the need for researchers conducting community engaged research to be sensitive to nomenclature and utilize terminology that the community they are engaging with prefers.

### 4.1 Limitations

Limitations of this study include variance in how co-design sessions occurred with a mixture of in-person focus groups and individual interviews, which may have impacted the participants' comfort in answering questions. However, group and individual interview design session length and content of participants' responses were comparable among design sessions. In accordance with person-and-family centered care and best practices for including people living with memory challenges in research, it is important to incorporate flexible options for participation to include their diverse lived experiences in research as they are often excluded (Griffith et al., 2023). Additionally, participants were not officially diagnosed with MCI or mild AD/ADRD and memory challenges were self-reported. Yet, MCI and mild AD/ADRD are often underdiagnosed in Hispanic/Latinos (Chen et al., 2019; Alzheimer's Association, 2023a). We included individuals who self-reported memory challenges as a result and limited inclusion with MoCA scores indicative of MCI and early AD/ADRD. Our sample was limited to Hispanic/Latino people from Ecuadorian, Mexican, and Puerto Rican origins in the Bronx, NY. Our findings should be interpreted with caution and not generalized as the program may need additional cultural adaptations before implementing with other Hispanic/Latino groups or geographic locations. While general cultural values may be analogous in other parts of the United States, tailoring would need to occur for local resources available in other Hispanic/Latino communities. Applying co-design enabled us to identify what aspects of the program and research design may require tailoring for other Hispanic/Latino groups in different geographic locations (e.g., preferred local green activities, delivery mode, and language). While co-design can be time-intensive, utilizing this approach allowed us to identify and tailor key aspects of the program and research design that mattered to participants from the beginning, which can promote engagement and uptake (Palmer et al., 2005). Co-design applied early in program development can guide other methods of community engagement once initial proof of concept has been established, such as utilizing a community advisory board, or informal conversations with community members to tailor the program to a new context.

### 4.2 Future directions

We provide lessons learned and recommendations for tailoring co-design sessions for Hispanic people living with memory challenges to inform future research (Table 1).

**Table 1:** Lessons learned and recommendations for tailoring co-design sessions for Hispanic/Latino people living with memory challenges and care partners.

**Lessons learned**	**Recommendations**
Importance of integrating cultural appropriateness in planning and conducting co-design sessions.	Surface level cultural appropriateness: •Create a research team with members who identify as Hispanic/Latino from the local community who are bilingual. •Ask team members to review the co-design topic guide and translated study materials to ensure language and common euphemisms are correct and culturally relevant. •Select a moderator and note taker who identify as Hispanic/Latino and are bilingual (Spanish and English). •Ask participants for preferred terminology for Hispanic/Latino groups and for discussing AD/ADRD and memory challenges. •Offer food and refreshments (ask for food restrictions and allergies ahead of time) that align with cultural preferences. Deep structure cultural appropriateness •Consider holding community discussions and events to provide education on brain health and signs of AD/ADRD prior to recruiting for co-design sessions to de-stigmatize cultural beliefs about AD/ADRD as normal aging and reduce feelings of helplessness and shame prior to co-design. •Create co-design sessions to align with cultural values of *personalismo* and *familismo* by tailoring topic guide content and offering options to allow people living with memory challenges to participate in co-design with a care partner. •Designate an ambassador or community health worker to welcome participants and answer questions before and during co-design sessions to foster *personalismo*. •Receive guidance from research team, community advisor, or a community advisory board on verbal and non-verbal communication during co-design sessions to honor cultural values of *respeto*, and *dignidad* (e.g., replacing looking away as a non-verbal cue to stop talking with a hand signal chosen by participants to promote *respeto*).
People living with memory challenges require flexible participation in co-design	•Hold co-design sessions at a location that is convenient for participants. •Ask if participants need special accommodations ahead of time (e.g., wheelchair access, audio, or visual accommodations). •Send guidelines for respectful communication and a topic guide 1–2 weeks prior to the design session. •Offer breaks. •Use simple language in the topic guide and study materials. •Include pictures of the moderator and study team in the topic guide to create a sense of familiarity and alleviate anxiety. •Consider creating a video or audio version of the topic guide for participants with visual impairments, low literacy, or difficulty reading due to cognitive decline. •Create a welcome video showing participants where they enter the building and where the session will be held so participants know what to expect. •Put up clear signage in the building to direct participants to the design session (e.g., large arrows, few words). •Ensure there is adequate lighting to prevent falls. •Avoid dim or overly bright lights. •Utilize pictures or visual aides to prompt discussion instead of words. •Minimize noise and visual distractions. •Speak slowly and clearly. •Provide added time for participants to respond to questions.
Participants living with varying social determinants of health may require additional flexibility for participation.	•Offer flexible scheduling. •Provide flexible options for participation (in-person, virtual). •Offer virtual options or in-home interviews for homebound participants.

More research is needed to understand which co-design methods (e.g., focus groups vs. individual interviews, group composition) are most effective with people living with memory challenges and their care partners and if there are variances among racial and ethnic groups and culture. Next steps include assessing feasibility and acceptability of the GAP for Hispanic/Latino people living with memory challenges in a single-arm feasibility pilot study. Community engaged researchers must include the perspectives of people living with memory challenges in their research to design effective interventions and maximize reach and uptake to eliminate health disparities. Researchers and can draw from this study and others (Galske et al., 2023) as examples to guide community-engaged research for this population.

## Data availability statement

The datasets presented in this article are not readily available because, per our IRB, access to raw interview data is restricted to research team members and our sponsor. Requests to access the datasets should be directed to: blassell@iu.edu.

## Ethics statement

The studies involving humans were approved by NYU Langone Institutional Review Board and the Indiana University Institutional Review Board. The studies were conducted in accordance with the local legislation and institutional requirements. The ethics committee/institutional review board waived the requirement of written informed consent for participation from the participants or the participants' legal guardians/next of kin because to support health equity as not all participants had access to or were proficient in technology. Informed consent was conducted electronically via Zoom, over the phone, and if possible, in-person. Written consent was received but we had a waiver of written consent in place to promote participation and health equity as we were recruiting from communities with lower resources. Written informed consent was obtained from the individual(s) for the publication of any potentially identifiable images or data included in this article.

## Author contributions

RL: Conceptualization, Data curation, Formal analysis, Funding acquisition, Investigation, Methodology, Resources, Validation, Visualization, Writing – original draft, Writing – review & editing. VT: Conceptualization, Writing – review & editing, Data curation, Formal analysis, Project administration, Validation. TP: Conceptualization, Writing – review & editing, Project administration, Investigation. MK: Conceptualization, Methodology, Resources, Writing – review & editing. JZ: Conceptualization, Writing – review & editing, Methodology, Resources, Supervision. LG: Conceptualization, Funding acquisition, Methodology, Supervision, Writing – review & editing, Resources. AB: Conceptualization, Funding acquisition, Methodology, Resources, Supervision, Writing – review & editing.
